# Complications of medial retropharyngeal, axillary, and ilio‐sacral lymphadenectomy in 127 dogs with malignant tumors

**DOI:** 10.1111/vsu.70003

**Published:** 2025-08-18

**Authors:** Luca Ciammaichella, Jessica Campanerut, Luciano Pisoni, Veronica Cola, Stefano Zanardi, Armando Foglia, Chiara Ferrari, Dina Guerra, Laura Marconato, Sara Del Magno

**Affiliations:** ^1^ Department of Veterinary Medical Sciences University of Bologna Bologna Italy

## Abstract

**Objective:**

To describe the complications of medial retropharyngeal, axillary, and ilio‐sacral lymphadenectomy in dogs with malignant tumors, and to identify possible risk factors.

**Study design:**

Retrospective cohort study.

**Animals:**

A total of 140 surgical accesses (86 axillary, 27 ilio‐sacral, 27 retropharyngeal) in 127 dogs.

**Methods:**

Dogs with cutaneous/subcutaneous cancer undergoing staging, lymph node (LN) mapping and extirpation of medial retropharyngeal, axillary, and/or ilio‐sacral LNs, with a minimum follow‐up of 1 month, were included. Retrieved information included signalment, tumor histotype, LN characteristics, excision of contiguous LNs, anesthesia duration, intra‐ and postoperative complications. Data were statistically analyzed to identify risk factors for complication development.

**Results:**

Intraoperative complications were registered in 3/140 (2%) procedures, including hemorrhage during medial iliac lymphadenectomy (2/27, 7%) and difficulty locating the axillary LN (1/86, 1%). Postoperative complications occurred in 32/140 (23%) cases, with rates of 41% (11/27) following ilio‐sacral lymphadenectomy, 26% (7/27) after medial retropharyngeal lymphadenectomy, and 16% (14/86) following axillary lymphadenectomy. Ilio‐sacral lymphadenectomy presented a higher risk of intraoperative (*p* = .033) and postoperative complications (*p* = .020). Enlarged (*p* = .030) or metastatic (*p* = .030) LNs were more prone to develop intraoperative complications. No risk factor retained significance on multivariate analysis. Median follow‐up, conducted through physical examination, was 225 days (range, 30–1735).

**Conclusion:**

Medial retropharyngeal and axillary lymphadenectomies were generally safe, associated with minor and easily manageable complications. Conversely, ilio‐sacral lymphadenectomy carried a higher risk of intraoperative complications, particularly in cases with enlarged LNs, and postoperative complications, potentially related to the caudal laparotomic approach.

**Clinical significance:**

Lymphadenectomies of medial retropharyngeal, axillary, and ilio‐sacral lymph nodes present relatively low complication rates.

## 1 INTRODUCTION

The sentinel lymph node (LN) is defined as the first LN that drains a cutaneous or subcutaneous tumor and its histopathologic evaluation is essential for identifying metastasis, serving both therapeutic and prognostic purposes.^1^, ^2^, ^3^ Thus, in recent years, lymphadenectomy has become more common alongside primary tumor excision. Moreover, the advancement and refinement of various techniques for sentinel LN mapping have enabled the detection and removal of LNs that are not readily accessible or typically palpable, including those located in the neck, axilla or body cavities.^4^ Intraoperative complications of lymphadenectomy are rare and often linked to the location of the LN, challenges in identification and potential damages to nearby anatomic structures. Although uncommon, hemorrhage stands out as one of the most frequently encountered intraoperative complications, usually resulting from accidental damage to the primary vasculature of the LN or nearby vessels.^5^, ^6^ Certain LNs, like those in the axilla or ilio‐sacral region, are often situated near critical vascular structures and are deeper within soft tissues or body cavities. Excising these LNs can pose greater challenges, potentially leading to prolonged surgical durations, unmanageable bleeding, or damage to brachial plexus nerves or iliac vessels.^6^, ^7^ Moreover, postoperative complications common to superficial lymphadenectomies, such as lymphoedema, seroma formation or incisional issues, may be more pronounced in the context of deep lymphadenectomies due to increased tissue manipulation, challenges in identifying the LN, larger incisions and the need to access body cavities.^6^ In humans, ilio‐sacral and pelvic lymphadenectomies are recognized as higher‐risk procedures in terms of postoperative lymphedema and incisional complications.^8^, ^9^ Due to the perceived higher risk of complications and more difficult surgeries, veterinary surgeons might hesitate to perform lymphadenectomies on LNs that are not readily accessible.^5^, ^10^

Thus, the aim of this retrospective research was to investigate the occurrence and characteristics of complications, along with potential risk factors, associated with the excision of medial retropharyngeal, axillary, and ilio‐sacral LNs in dogs diagnosed with cutaneous/subcutaneous malignant tumors.

## 2 MATERIALS AND METHODS

### 2.1 Medical record search

The hospital electronic medical record database (Fenice 04.70. KA10; ZakSoft S.r.l., Bologna, Italy) of the University Veterinary Hospital of Bologna was retrospectively reviewed from July 2017 to July 2023. Dogs diagnosed with cutaneous or subcutaneous cancer that underwent complete staging, preoperative LN mapping and extirpation of medial retropharyngeal, axillary, and/or ilio‐sacral LNs, with at least 1 month of postoperative follow‐up, were included. Dogs undergoing lymphadenectomy for other reasons, those with significant comorbidities or distant metastases, and those with incomplete medical records (i.e., insufficient or missing data in the electronic database) were excluded from the study.

Information obtained from the medical records included signalment (i.e., breed, sex, neutering status, age, bodyweight, body condition score [BCS]), primary tumor histotype, use of intraoperative mapping technique, location, number, and size of the excised LNs, any concurrent excision of adjacent LNs, duration of anesthesia, surgical complications and histologic LN status. Ilio‐sacral lymph center referred to the group of medial iliac, internal iliac and sacral LNs.

### 2.2 Outcomes

Complications related to medial retropharyngeal, axillary, and ilio‐sacral lymphadenectomies were recorded and graded according to the Common Terminology Criteria for Adverse Events (VCOG‐CTCAE version 2).^11^ Briefly, intraoperative complications, spanning from skin incision to closure, were categorized into five grades: grade 1 (requiring no or minimal additional treatment/intervention, without further consequence), grade 2 (requiring additional treatment/intervention, without further consequence), grade 3 (requiring additional treatment/intervention, with further non‐life‐threatening consequence), grade 4 (requiring additional treatment/intervention, with further life‐threatening consequence) and grade 5 (resulting in death). Postoperative complications were categorized as short‐term, occurring from skin closure up to 15 days postoperatively, and long‐term, arising beyond 15 days. Complications were subsequently graded as follows: grade 1 (asymptomatic, requiring only observation or topical treatment), grade 2 (symptomatic, requiring local care and/or systemic medical therapy), grade 3 (symptomatic, requiring invasive intervention or reoperation), grade 4 (life‐threatening, requiring urgent invasive intervention or reoperation) and grade 5 (resulting in death).^11^

The follow‐up time was determined by calculating the number of days from surgery to either the patient's death or the date of the last clinical recheck.

After surgical resection, all specimens underwent routine histologic processing and evaluation performed by a board‐certified veterinary pathologist.

### 2.3 Statistical analysis

Collected data were summarized through descriptive statistics and analyzed through medical statistical software (MedCalc Software Ltd., Ostend, Belgium). Each surgical access to the LNs was treated as an event. The population was divided into three groups based on the type of lymphadenectomy: axillary, medial retropharyngeal and ilio‐sacral. Continuous data were tested for normality using the D'Agostino and Pearson omnibus normality test. Normally distributed data are summarized as mean (SD) and analyzed via parametric tests, while not normally distributed data are presented as median (range) and were analyzed using non‐parametric tests. Categorical data are reported as frequencies and percentages. To identify risk factors, *χ* ^2^ tests were used to compare intra‐ and postoperative complication rates with categorical variables (e.g., breed, sex, neuter status, primary tumor histotype, concurrent excision of superficial contiguous LNs, additional surgical procedures, use of intraoperative methylene blue mapping, and histopathologic nodal status), while the Mann–Whitney U test was employed to compare complications with continuous variables (e.g., age, weight, BCS, number and size of excised LN, anesthesia time). Multivariate logistic regression was also performed, incorporating all variables with a univariate *p* < .2. The tumor‐specific nodal metastatic rate was calculated as the number of metastatic nodes out of the total number of LNs for each histotype. The overall mortality rate was calculated based on the number of patients deceased during the study period, while the median survival time (MST) was determined for those who died for tumor‐related causes. Statistical significance was set at *p* ≤ .05.

## 3 RESULTS

### 3.1 Demographics

Overall, 127 dogs were included. There were 86/127 (68%) pure‐breed dogs (Labrador Retriever, *n* = 10; Golden Retriever, *n* = 9; Boxer, *n* = 7; English Setter, *n* = 7; French Bulldog, *n* = 7; Dachshund, *n* = 6; others, *n* = 40) and 41/127 (32%) mixed‐breed dogs. A total of 73 of 127 (58%) dogs were females (spayed, *n* = 53; intact, *n* = 20) and 54/127 (43%) were males (castrated, *n* = 29; intact, *n* = 25). The median age was 9 years (range, 1–18) and the median weight was 21 kg (range, 1.5–65). Body condition score was available for 83/127 (65%) dogs and median value was 5 (range, 1–8) (Table 1).

**TABLE 1:** Demographics and tumor histotype.

Dogs	127
Breed	
Mixed breed	41/127 (32%)
Labrador Retriever	10/127 (8%)
Golden Retriever	9/127 (7%)
Boxer	7/127 (6%)
English Setter	7/127 (6%)
French Bulldog	7/127 (6%)
Dachshund	6/127 (5%)
American Staffordshire Terrier	2/127 (2%)
Beagle	2/127 (2%)
Czechoslovakian Wolf	2/127 (2%)
Dogo Argentino	2/127 (2%)
Italian Hound	2/127 (2%)
Maltese	2/127 (2%)
Miniature Pinscher	2/127 (2%)
Poodle	2/127 (2%)
Pug	2/127 (2%)
Weimaraner	2/127 (2%)
Akita Inu	1/127 (1%)
Appenzeller Sennenhund	1/127 (1%)
Australian Shepherd	1/127 (1%)
Bernese Mountain Dog	1/127 (1%)
Border Collie	1/127 (1%)
Cane Corso	1/127 (1%)
Chihuahua	1/127 (1%)
Dobermann	1/127 (1%)
German Shepherd	1/127 (1%)
Great Dane	1/127 (1%)
Griffon Bleu de Gascogne	1/127 (1%)
Jack Russel Terrier	1/127 (1%)
Newfoundland	1/127 (1%)
Pitbull	1/127 (1%)
Rottweiler	1/127 (1%)
Shar Pei	1/127 (1%)
Shiba Inu	1/127 (1%)
Spanish Breton	1/127 (1%)
Springer Spaniel	1/127 (1%)
Staffordshire Bull Terrier	1/127 (1%)
Age (y)	9 (1–18)
Weight (kg)	21 (1.5–65)
BCS (1–9)^a^	5 (1–8)
Sex	
FS	53/127 (42%)
MC	29/127 (23%)
M	25/127 (20%)
F	20/127 (16%)
Tumor histotype	
MCT	83/127 (65%)
Carcinoma	28/127 (22%)
Melanoma	8/127 (6%)
STS	6/127 (5%)
Plasma cell tumor	2/127 (2%)

*Note*: Categorical data are expressed with frequencies (percentages), continuous data are reported with median (range).

Abbreviations: BCS, body condition score; F, female; FS, spayed female; M, male; MC, castrated male; MCT, mast cell tumor; STS, soft tissue sarcoma; y, years.

^a^ Available in 83/127 dogs.

### 3.2 Staging work‐up and primary tumor histotype

Staging work‐up included complete bloodwork, urinalysis, cytologic evaluation of the primary tumor, thoracic radiographs and abdominal ultrasound or total‐body computed tomography (CT), and sentinel LN mapping by means of CT‐lymphography (107/127, 84%) or indirect lymphangiography (20/127, 16%) with a non‐ionic iodinate contrast medium. If the sentinel LN was not identified during mapping (15/127, 12%), the regional LN was selected based on the lymphosome model.^12^ In cases of mast cell tumors (MCTs), fine‐needle aspiration and subsequent cytologic assessment of liver and spleen were also performed. Primary tumor histotype included 83/127 (65%) MCTs, 28/127 (22%) carcinomas, 8/127 (6%) melanomas, 6/127 (5%) soft tissue sarcomas, and 2/127 (2%) plasma cell tumors (Table 1).

### 3.3 Intraoperative LN mapping

In cases of sentinel LN intraoperative mapping, methylene blue was injected peritumorally, after aseptic preparation of the surgical field, at a volume of 0.1 mL per quadrant across all four quadrants, as previously described.^1^ This procedure was performed in 82/140 (59%) accesses, with no differences in the timing (120 min; range, 60–360, for both groups; *p* = .290). However, a slightly reduced rate of intraoperative (0/66, 0% vs. 3/74, 4%–*p* = .141) and postoperative (13/66, 20% vs. 19/74, 26%–*p* = .918) complications was observed in cases where methylene blue was used compared to those where it was not (Table 2).

**TABLE 2:** Overall and group‐specific distribution of variables.

Group	Overall (140)	Axillary (86)	Medial retropharyngeal (27)	Ilio‐sacral (27)
Intraoperative complications	3/140 (2%)	1/86 (1%)	0/27 (0%)	2/27 (7%)
Postoperative complications	32/140 (23%)	14/86 (16%)	7/27 (26%)	11/27 (41%)
Number of excised LNs	One 134/140 (96%)	One 81/86 (94%)	One 26/27 (96%)	One 27/27 (100%)
	Two 6/140 (4%)	Two 5/86 (6%)	Two 1/27 (4%)	
Size of excised LNs (cm)	2 (0.2–6.5)	1.6 (0.2–5.0)	2.8 (0.2–6.5)	2.0 (0.2–6.0)
Anesthesia time (min)	120 (60–360)	120 (60–360)	150 (60–320)	140 (90–350)
Concurrent excision of superficial LNs	Yes 87/140 (62%)	Yes 44/86 (51%)	Yes 24/27 (89%)	Yes 19/27 (70%)
	No 53/140 (38%)	No 42/86 (49%)	No 3/27 (11%)	No 8/27 (30%)
Further surgical procedures performed	Yes 84/140 (60%)	No 47/86 (55%)	Yes 24/27 (89%)	Yes 21/27 (78%)
	No 56/140 (40%)	Yes 39/86 (45%)	No 3/27 (11%)	No 6/27 (22%)
Use of methylene blue	No 82/140 (59%)	No 49/86 (57%)	No 20/27 (74%)	Yes 14/27 (52%)
	Yes 58/140 (41%)	Yes 37/86 (43%)	Yes 7/27 (26%)	No 13/27 (48%)
Histopathologic nodal status	Non‐metastatic 88/140 (63%)	Non‐metastatic 57/86 (66%)	Non‐metastatic 15/27 (56%)	Non‐metastatic 16/27 (59%)
	Metastatic 52/140 (37%)	Metastatic 29/86 (34%)	Metastatic 12/27 (44%)	Metastatic 11/27 (41%)
Follow‐up (days)	225 (30–1735)	211 (30–1735)	225 (29–791)	312 (30–746)

*Note*: Categorical data are expressed with frequencies (percentages), continuous data are reported with median (range).

Abbreviations: LN, lymph node; min, minutes.

### 3.4 Surgical accesses and LN characteristics

In total 140 surgical approaches for lymphadenectomies were included because bilateral lymphadenectomy, overall performed in 29/127 dogs (23%), were performed via a bilateral access in the axillary group (*n* = 9), while through the same median access for medial iliac (*n* = 14) and medial retropharyngeal (*n* = 6) LNs. Also, 4/127 (3%) dogs had multiple LNs removed from different lymph centers (axillary and medial retropharyngeal, *n* = 2; axillary and bilateral medial iliac, *n* = 2) (Table 2).

A total of 86 out of 140 (61%) surgical accesses were performed for axillary LNs, via a longitudinal incision in the caudal axillary region, either on dorsal recumbency with extended shoulders, or on lateral recumbency with the thoracic limb of the upper side abducted, based on the surgeon's preference and on the location of the primary tumor to be excised. Accessory axillary LNs were also excised in 7/86 (8%) accesses. A total of 27 out of 140 (19%) surgical accesses were performed for medial retropharyngeal LNs, via a ventral cervical cranial midline approach on dorsal recumbency. A total of 27 out of 140 (19%) surgical accesses were performed for ilio‐sacral LNs, via a caudal celiotomy approach on dorsal recumbency. Within the surgical access, the left and/or right medial iliac LNs were excised in all cases. Additionally, sacral LNs were excised in 9/27 (33%) accesses, and internal iliac LNs were excised in 4/27 (15%) accesses. In seven out of 140 (5%) accesses, multiple LNs were excised from the same lymph center. Specifically, two LNs were removed in 6/140 (4%) accesses (axillary, *n* = 5; medial retropharyngeal, *n* = 1) and three sacral LNs were removed in 1/140 (1%) access. Overall, 188 LNs were excised (Table 3). In 99 (71%) of the 140 surgical accesses, a superficial LN near or intersecting with the incision site was also excised, either based on preoperative mapping or due to the concurrent excision of other tumors along with their corresponding sentinel LNs. Specifically, superficial cervical LNs were also excised during 39/86 (45%) accesses for axillary LNs, mandibular LNs were also excised during 24/27 (89%) accesses for medial retropharyngeal LNs, and superficial inguinal LNs were also excised during 19/27 (70%) accesses for ilio‐sacral LNs.

**TABLE 3:** Number of LNs excised.

LN excised		Unilateral/median	Bilateral	Multiple LNs^a^	Total LNs
Axillary	Main	63	9	5 (2 LNs)	**91**
	Accessory	7			**7**
Ilio‐sacral	Medial iliac	13	14		**41**
	Sacral	8		1 (3 LNs)	**11**
	Internal iliac	4			**4**
Medial retropharyngeal		20	6	1 (2 LNs)	**34**
Overall					**188**

Abbreviation: LN, lymph node.

^a^ Number of cases with multiple LNs per lymph center (number of LNs per lymph center).

The median maximum diameter of axillary, medial retropharyngeal, and ilio‐sacral LNs was, respectively, 1.6 cm (range, 0.2–5.0), 2.8 cm (range, 0.2–6.5), and 2.0 cm (range, 0.2–6.0). Bipolar electrosurgery was used for hemostasis if necessary. Additional surgical procedures (i.e., ovariectomy, orchiectomy, preventive gastropexy), apart from primary tumor excision, were concurrently performed during 86/140 (61%) accesses. The median duration of the anesthetic procedure for axillary, medial retropharyngeal, and ilio‐sacral lymphadenectomy was respectively 120 min (range, 60–360), 150 min (range, 60–320), and 140 min (range, 90–350). Postoperative hospitalization and medications were determined based on the preferences of the surgeon and anesthetist, with analgesia ensured in all cases using opioids and/or non‐steroidal anti‐inflammatory drugs.

### 3.5 Intraoperative complications

Intraoperative complications were registered in 3/140 (2%) accesses (Table 4). In 2/27 (7%) cases, grade 2 hemorrhage occurred during enlarged medial iliac LN dissection, resulting from accidental rupture of the LN vasculature and accidental damage to one iliac vein, respectively. In both instances, bleeding was successfully managed through electrocautery or ligation of the affected vessels. In the third case (1/86, 1%), the axillary LN could not be identified during routine surgical exploration (grade 2), requiring prompt intraoperative ultrasonography of the region to locate the LN. In the ilio‐sacral group, univariate analysis revealed that an increase in maximum diameter was associated with a higher risk of intraoperative complications (i.e., bleeding) (*p* = .030).

**TABLE 4:** Overall and group‐specific intra‐ and postoperative complications.

Group	Intraoperative complications				Postoperative complications			
	Grade 1	Grade 2	Grade 3	Overall	Grade 1	Grade 2	Grade 3	Overall
Axillary (86)	0/86 (0%)	1/86 (1%)	0/86 (0%)	1/86 (1%)	13/86 (15%)	1/86 (1%)	0/86 (0%)	14/86 (16%)
Ilio‐sacral (27)	0/27 (0%)	2/27 (7%)	0/27 (0%)	2/27 (7%)	7/27 (26%)	3/27 (11%)	1/27 (4%)	11/27 (41%)
Medial retropharyngeal (27)	0/27 (0%)	0/27 (0%)	0/27 (0%)	0/27 (0%)	6/27 (22%)	1/27 (4%)	0/27 (0%)	7/27 (26%)
Overall (140)	0/140 (0%)	3/140 (2%)	0/140 (0%)	3/140 (2%)	26/140 (19%)	5/140 (4%)	1/140 (1%)	32/140 (23%)

*Note*: Categorical data are expressed with frequencies (percentages).

### 3.6 Postoperative complications

Overall, postoperative complications arose in 32/140 (23%) accesses (Table 4).

**FIGURE 1:**
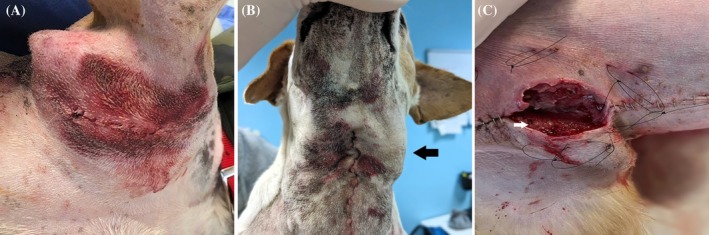
Postoperative complications of specific lymphadenectomies. (A) Grade 1 wound hematoma after left axillary lymphadenectomy. (B) Grade 1 neck edema after left medial retropharyngeal lymphadenectomy; note the greater edema on the left side (black arrow). (C) Grade 3 laparotomy wound dehiscence and infection—after surgical debridement—developed after ilio‐sacral lymphadenectomy; note the granulation tissue (white arrow) and the pre‐placed loops for tie‐over medications.

Among these, 14/86 (16%) complications were documented in axillary accesses. Grade 1 complications occurred in 13/14 (93%) cases, encompassing subcutaneous seroma (*n* = 6); thoracic limb edema (*n* = 5); wound hematoma (*n* = 1) (Figure 1A); and both thoracic limb edema and wound hematoma (*n* = 1). A grade 2 complication was recorded in 1/14 (7%) access, manifesting as a superficial wound infection that required both topical and systemic antibiotic treatment. In 6/14 (43%) of these accesses, a superficial cervical lymphadenectomy was also performed, with no difference (*p* = .519) in complication rates compared to those that did not undergo this procedure (8/47, 17%). In 2/14 (14.3%) accesses, the accessory axillary LN was also excised; 2/14 (14%) accesses were bilateral. Seven out of 27 (26%) dogs experienced complications of the medial retropharyngeal LNs access. Specifically, grade 1 complications occurred in 6/7 (86%) accesses, consisting of neck and face edema (*n* = 4) (Figure 1B) and subcutaneous seroma (*n* = 2). A grade 2 complication was documented in 1/7 (14%) accesses, manifesting as a superficial wound infection that required topical medication (honey), bandages and a short course (i.e., 3–5 days) of systemic antimicrobic therapy. Mandibular lymphadenectomy was also performed in 7/7 (100%) that underwent this approach, while no complications were observed in dogs not subjected to the procedure (*p* = .372). Three out of these seven (43%) accesses were used for bilateral medial retropharyngeal lymphadenectomy.

A total of 11 of the 27 (41%) accesses for ilio‐sacral lymphadenectomy developed complications. Specifically, grade 1 complications occurred in 7/11 (64%) accesses, encompassing pelvic limb edema (*n* = 3), subcutaneous seroma at the laparotomy wound (*n* = 2), inguinal erythema (*n* = 1), and laparotomy wound hematoma (*n* = 1). Grade 2 complications were reported in 3/11 (27%) accesses, including partial cutaneous wound dehiscence (*n* = 2) and superficial wound infection (*n* = 1), both requiring topical ointments (honey and hyaluronic acid ointments), tie‐over bandages and systemic antimicrobic therapy. In 1/11 (9%) accesses, a grade 3 complete laparotomy wound dehiscence and infection occurred, requiring revision surgery, debridement and direct resuturing of the defect, and systemic antimicrobic therapy based on bacterial culture and sensitivity test (Figure 1C). During 8 (73%) of these 11 accesses, a superficial inguinal lymphadenectomy was also performed, with no difference (*p* = .741) in complication rates compared to those not receiving it (3/8, 38%). In 6/11 (55%) accesses the medial iliac was excised bilaterally.

### 3.7 Histopathology

Based on histopathology, 57 out of 188 (30%) LNs were metastatic, including 28/98 (29%) axillary (main or accessory), 11/34 (32%) medial retropharyngeal and 18/56 (32%) ilio‐sacral. The tumor‐specific nodal metastatic rate was 46% for MCTs (38/83), 32% for carcinomas (9/28), 25% for melanomas (2/8), 0% for plasma cell tumors (0/2) and soft tissue sarcomas (0/6). Overall, a metastatic LN on histopathology appeared to be more prone to intraoperative complications (*p* = .030).

### 3.8 Follow‐up

The median follow‐up time was 225 days (range, 30–1735). Follow‐up was performed through physical examination in 127/127 patients (100%). The median follow‐up for dogs that developed postoperative complications was longer than for those that did not, both overall (323 days; range, 30–1735, vs. 323 days; range, 30–1735; *p* = .026) and within the axillary lymphadenectomy group (330 days; range, 30–1735, vs. 154 days; range, 30–903; *p* = .032). A total of 20 (16%) of the 127 dogs died during the study period, due to tumor‐related (18/20, 90%) or unrelated (2/20, 10%) causes. Notably, no deaths occurred due to surgical complications, with a MST of 374 days.

### 3.9 Risk factors analysis

Ilio‐sacral lymphadenectomy was associated with a higher risk of intraoperative complications (*p* = .033) when compared to the overall population, and with a higher risk of postoperative complications compared to the other groups (*p* = .047), particularly compared to axillary lymphadenectomy (*p* = .008) and the overall population (*p* = .020). In contrast, axillary lymphadenectomy appeared to be less prone to postoperative complications compared to the overall population (*p* = .034). An increased maximum diameter of ilio‐sacral LNs (*p* = .030) and the presence of metastases on histopathology (*p* = .030) were associated with the development of intraoperative complications. For postoperative complications, an association was found with a longer follow‐up, both overall (*p* = .026) and in the axillary group (*p* = .032) (Table 5). However, no identified risk factor retained significance on multivariate analysis (Table 6).

**TABLE 5:** Univariate analysis of risk factors for intra‐ and postoperative complications overall and within groups.

Group	Axillary		Medial retropharyngeal		Ilio‐sacral		Overall	
Variable	Intraoperative complications (1/86)	Postoperative complications (14/86)	Intraoperative complications (0/27)	Postoperative complications (7/27)	Intraoperative complications (2/27)	Postoperative complications (11/27)	Intraoperative complications (3/140)	Postoperative complications (32/140)
Breed	French Bulldog 1/1 (100%) *p* = .973	Labrador Retriever 3/14 (21%) Mixed breed 2/14 (14%) Appenzeller Sennenhund 1/14 (7%) Beagle 1/14 (7%) Cane Corso 1/14 (7%) Dachshund 1/14 (7%) Golden Retriever 1/14 (7%) Pitbull 1/14 (7%) Pug 1/14 (7%) Shiba Inu 1/14 (7%) Weimaraner 1/14 (7%) *p* = .182		Mixed breed 6/7 (86%) Boxer 1/7 (14%) *p* = .131	Dachshund 1/2 (50%) Mixed breed 1/2 (50%) *p* = .263	Mixed breed 5/11 (45%) English Setter 2/11 (18%) Beagle 1/11 (9%) Boxer 1/11 (9%) Czechoslovakian Wolf 1/11 (9%) Weimaraner 1/11 (9%) *p* = .464	Dachshund 1/3 (33%) French Bulldog 1/3 (33%) Mixed breed 1/3 (33%) *p* = .953	Mixed breed 13/32 (41%) Boxer 2/32 (6%) Labrador Retriever 3/32 (9%) Beagle 2/32 (6%) English Setter 2/32 (6%) Weimaraner 2/32 (6%) Appenzeller Sennenhund 1/32 (3%) Czechoslovakian Wolf 1/32 (3%) Cane Corso 1/32 (3%) Dachshund 1/32 (3%) Golden Retriever 1/32 (3%) Pitbull 1/32 (3%) Pug 1/32 (3%) Shiba Inu 1/32 (3%) *p* = .429
Sex and neuter status	M 1/1 (100%) *p* = .183	F 5/14 (36%) FS 5/14 (36%) MC 3/14 (21%) M 1/14 (7%) *p* = .503		M 3/7 (43%) MC 2/7 (29%) FS 2/7 (29%) *p* = .629	MC 2/2 (100%) *p* = .071	MC 5/11 (45%) FS 3/11 (27%) F 2/11 (18%) M 1/11 (9%) *p* = .188	M 1/3 (33%) MC 2/3 (67%) *p* = .962	MC 10/32 (31%) FS 10/32 (31%) F 7/32 (22%) M 5/32 (16%) *p* = .860
Age (y)	5 NA	9 (5–12) *p* = .482		11 (5–14) *p* = .694	12 (9–15) *p* = .292	10 (5–15) *p* = .564	9 (5–15) *p* = .994	9 (−15) *p* = .841
Weight (kg)	12.6 NA	26.9 (6.2–54.5) *p* = .663		23.2 (6.7–43.5) *p* = .717	12 (5.4–18.7) *p* = .161	22.4 (8.9–65.0) *p* = .913	12.6 (5.4–18.7) *p* = .102	23.1 (6.2–65.0) *p* = .569
BCS (1–9)^a^	5 NA	4 (3–6) *p* = .092		4 (4–6) *p* = .713	4 (3–5) *p* = .224	5 (3–7) *p* = .214	5 (3–5) *p* = .450	4 (3–7) *p* = .074
Intraoperative complications	NA	No 14/14 (100%) *p* = .631		No 7/7 (100%) *p* = .060	NA	No 10/11 (91%) Yes 1/11 (9%) *p* = .819	NA	No 31/32 (97%) Yes 1/32 (3%) *p* = .730
Postoperative complications	No 1/1 (100%) *p* = .631	NA		NA	No 1/2 (50%) Yes 1/2 (50%) *p* = .819	NA	No 2/3 (67%) Yes 1/3 (33%) *p* = .730	NA
Number of excised LNs	One 1/1 (100%) *p* = .791	One 12/14 (86%) Two 2/14 (14%) *p* = .191		One 6/7 (86%) Two 1/7 (14%) *p* = .093	One 2/2 (100%) *p* = .348	One 11/11 (100%) *p* = .868	Oone 3/3 (100%) *p* = .957	One 29/32 (91%) Two 3/32 (9%) *p* = .177
Size of excised LNs (cm)	0.7 NA	2.0 (0.5–4.0) *p* = .444		2.5 (0.2–6.5) *p* = .524	5.5 (5.0–6.0) ***p* = .030**	2.0 (1–5‐5.0) *p* = .658	5.0 (0.7–6.0) *p* = .273	2.0 (0.3–6.5) *p* = .378
Anesthesia time (min)	120 NA	135 (90–360) *p* = .230		180 (150–300) *p* = .257	240 (180–300) *p* = .087	150 (90–350) *p* = .979	180 (120–300) *p* = .259	150 (90–360) *p* = .081
Primary tumor histotype	MCT 1/1 (100%) *p* = .949	MCT 11/14 (79%) Carcinoma 2/14 (14%) STS 1/14 (7%) *p* = .755		MCT 3/7 (43%) Melanoma 3/7 (43%) Carcinoma 1/7 (14%) *p* = .973	Carcinoma 2/2 (100%) *p* = .959	MCT 8/11 (73%) Carcinoma 3/11 (27%) *p* = .734	Carcinoma 2/3 (67%) MCT 1/3 (33%) *p* = .456	MCT 22/32 (69%) Carcinoma 6/32 (19%) Melanoma 3/32 (9%) STS 1/32 (3%) *p* = .766
Concurrent excision of superficial LNs	Yes 1/1 (100%) *p* = .320	No 8/14 (57%) Yes 6/14 (43%) *p* = .519		Yes 7/7 (100%) *p* = .372	No 1/2 (50%) Yes 1/2 (50%) *p* = .540	Yes 8/11 (73%) No 3/11 (27%) *p* = .741	Yes 2/3 (67%) No 1/3 (33%) *p* = .880	Yes 21/32 (66%) No 11/32 (34%) *p* = .702
Further surgical procedures performed	No 1/1 (100%) *p* = .371	Yes 8/14 (57%) No 6/14 (43%) *p* = .279		Yes 6/7 (86%) No 1/7 (14%) *p* = .826	No 1/2 (50%) Yes 1/2 (50%) *p* = .269	Yes 9/11 (82%) No 2/11 (8%) *p* = .840	No 2/3 (67%) Yes 1/3 (33%) *p* = .343	Yes 23/32 (72%) No 9/32 (28%) *p* = .162
Use of methylene blue	No 1/1 (100%) *p* = .383	No 9/14 (64%) Yes 5/14 (36%) *p* = .551		No 5/7 (71%) Yes 2/7 (29%) *p* = .908	No 2/2 (100%) *p* = .552	Yes 6/11 (55%) No 5/11 (45%) *p* = .821	No 3/3 (100%) *p* = .141	No 19/32 (59%) Yes 13/32 (41%) *p* = .918
Histopathologic nodal status	Metastatic 1/1 (100%) *p* = .183	Metastatic 7/14 (50%) Non‐metastatic 7/14 (50%) *p* = .241		Non‐metastatic 4/7 (57%) Metastatic 3/7 (43%) *p* = .973	Metastatic 2/2 (100%) *p* = .315	Non‐metastatic 8/11 (73%) Metastatic 3/11 (27%) *p* = .098	Metastatic 3/3 (100%) ***p* = .030**	Non‐metastatic 19/32 (59%) Metastatic 13/32 (41%) *p* = .950
Follow‐up (days)	220 NA	330 (30–1735) ***p* = .032**		225 (64–728) *p* = .665	179 (99–258) *p* = .564	379 (30–746) *p* = .795	220 (99–259) *p* = .747	323 (30–1735) ***p* = .026**

*Note*: Categorical data are expressed with frequencies (percentages), continuous data are reported with median (range). Significant *p*‐values are in bold.

Abbreviations: BCS, body condition score; F, female; FS, spayed female; LN, lymph node; M, male; MC, castrated male; MCT, mast cell tumor; min, minutes; NA, not applicable; STS, soft tissue sarcoma; y, years.

^a^ Available in 83/127 dogs.

**TABLE 6:** Multivariate analysis of risk factors for intra‐ and postoperative complications overall and within groups.

Group	Variables	*p‐*value
Axillary		
Intraoperative complications	Sex and neuter status	.997
	Histopathologic nodal status	.997
Postoperative complications	Breed	.998
	BCS^a^	.062
	Number of LNs excised	.123
	Follow‐up	.084
Medial retropharyngeal		
Intraoperative complications	NA	
Postoperative complications	Breed	.997
	Intraoperative complications	.997
	Number of LNs excised	.124
Ilio‐sacral		
Intraoperative complications	Sex and neuter status	.999
	Weight	.999
	Size of excised LNs	.998
	Anesthesia time	.999
Postoperative complications	Sex and neuter status	.601
	Histopathologic nodal status	.215
Overall		
Intraoperative complications	Weight	.109
	Use of methylene blue	.995
	Histopathologic nodal status	.994
Postoperative complications	BCS^a^	.059
	Number of LNs excised	.254
	Anesthesia time	.064
	Further surgical procedures performed	.208
	Follow‐up	.134

*Note*: Significant *p*‐values are in bold.

Abbreviations: BCS, body condition score; LN, lymph node; NA, not applicable.

^a^ Available in 83/127 dogs.

## 4 DISCUSSION

A relatively low intra‐ and postoperative complication rate was documented after extirpation of medial retropharyngeal, axillary, and/or ilio‐sacral LNs, with most complications being self‐limiting or manageable with minor medical intervention. This percentage was comparable to those reported in the veterinary literature for superficial lymphadenectomies (10%–29%).^13^, ^14^, ^15^, ^16^

Intraoperative complications were infrequent and primarily associated with the previously reported risks of bleeding and challenges in LN identification. Bleeding was a rare occurrence, significantly more common during medial iliac lymphadenectomy (4% of ilio‐sacral lymphadenectomies), which was comparable to or even less frequent than the prevalence reported in previous studies (5.3%–17.6%).^17^, ^18^, ^19^, ^20^ This intraoperative complication may potentially be life‐threatening, leading to conditions such as hypotension, uncontrollable bleeding or even death. Additionally, it may impact quality of life by causing paraparesis or urinary incontinence.^17^, ^18^, ^19^, ^20^ However, in our cases, bleeding was moderate and managed without sequelae. The low severity of intraoperative complications may have been linked to the size of the LNs. In most cases, we primarily removed LNs of normal size, which likely contributed to the low rate of intraoperative complications. It is acknowledged that LN size can influence the development of complications.^6^, ^19^, ^20^ In fact, both dogs that experienced intraoperative hemorrhage had enlarged medial iliac LNs, and the increased size of these LNs was significantly associated with a higher risk of intraoperative complications, despite the low sample size. Additionally, the challenging identification of an axillary LN in one case required intraoperative ultrasonographic guidance, which resulted in an extended surgical time. Although infrequent, this may result from a limited understanding of anatomy, surgeon inexperience or variations in individual anatomy. Recent reports underscore the importance of employing a precise surgical technique based on anatomical knowledge, especially when dealing with LNs that are not readily accessible.^10^, ^21^ The use of anchor‐wire needles is considered a valuable option for LN localization, as it reduces tissue trauma and shortens surgical times associated with LN identification, particularly in deep lymphadenectomies.^15^ This technique could have been beneficial in our cases as well. Furthermore, intraoperative dye mapping and/or guidance, in addition to its role in sentinel LN mapping, could help alleviate identification challenges. Methylene blue and indocyanine green highlight the LN contours and the lymphatic vessels, thereby reducing both surgical and anesthesiologic times during lymphadenectomies, especially in challenging or unconventional cases.^1^, ^13^, ^15^, ^22^, ^23^, ^24^ Notably, intraoperative complications occurred only in cases where intraoperative dye was not used, while postoperative complications were slightly reduced in those cases where dye was applied. However, no significant difference was found in terms of surgical duration or the development of complications between dogs that received methylene blue for intraoperative LN mapping and those that did not. It is possible that the relatively limited use of methylene blue in this study sample may have prevented us from identifying significant results.

The overall postoperative complication rate in our population (23%) aligned with those reported in the existing literature (10%–29%).^13^, ^14^, ^15^, ^16^, ^25^ Most complications were mild and self‐limiting (grade 1%–81%), primarily characterized by regional edema (50%) and wound seroma (38%), most likely stemming from the compromised drainage function of the lymphatic system.^13^, ^14^, ^15^, ^16^, ^25^ When comparing our findings on specific lymphadenectomies with other studies, rates for axillary and medial retropharyngeal lymphadenectomies were consistent with previous descriptions.^13^, ^14^, ^15^, ^16^, ^25^ However, ilio‐sacral LN removal showed a significantly higher postoperative complication rate (41%) compared to other lymphadenectomies in our population and previous literature (0%–10.4%).^6^, ^13^, ^19^ Also, ilio‐sacral LN removal was associated with a unique grade 3 incisional complication necessitating surgical revision. Notably, in human medicine, the complication rate for ilioinguinal lymphadenectomy is high (22%–52%).^8^, ^26^ Significant risk factors for secondary lymphedema and incisional complications after ilioinguinal lymphadenectomy in humans include obesity and increased body mass index. Moreover, lymphedema appears to predispose individuals to the development of further incisional complications, such as surgical site infections and seromas.^27^, ^28^, ^29^ In the case of concurrent pelvic lymphadenectomy, the rate of complications further increased.^9^, ^27^, ^29^

In dogs, the increased risk of incisional complications may be intrinsic to the surgical approach for several reasons. First, caudal laparotomy could theoretically pose a higher risk due to the presence of inguinal fat, which may become inflamed and develop mild edema or even steatonecrosis due to surgical manipulation. Second, the caudal abdominal and inguinal region's anatomical characteristics, such as the presence of mammary glands or the prepuce, create a moist environment postoperatively and are more susceptible to patient self‐trauma if proper restraint, like an E‐collar or medical pet shirt, is not ensured. Third, accessing dorsal areas of the peritoneal cavity and manipulating firm ilio‐sacral LNs may result in compressing or stretching of cutaneous and subcutaneous tissues, exacerbating surgical trauma. Lastly, while not conclusively confirmed by our survey, concurrent extirpation of superficial LNs, such as superficial inguinal ones, might increase the risk of postoperative inflammation and edema due to additional or prolonged surgical manipulation and further disruption of lymphatic drainage. Unfortunately, BCS, a suggested risk factor from human medicine (e.g., obesity, body mass index), was not significantly associated to the development of complications in our survey, also because it was only available for a part of the population. These findings, coupled with intraoperative bleeding, warrant consideration when contemplating intracavitary ilio‐sacral lymphadenectomy and should be discussed with owners, particularly in oncologic patients where surgical complications may delay adjuvant treatments.^6^, ^30^ Novel intraoperative mapping techniques and sentinel node navigation surgery have shown promise in reducing complication rates in humans, especially in pelvic lymphadenectomy.^8^ Additionally, a laparoscopic approach may decrease the incidence of postoperative complications in both human and veterinary surgery.^17^, ^18^, ^29^, ^31^, ^32^

Concurrent removal of superficial LNs was not associated with an increased risk of postoperative complications. In cases of axillary LNs removal, less than half (43%) of dogs experiencing postoperative complications also underwent superficial cervical lymphadenectomy, although the complication rate was lower (16%) for those that did not receive it. Thus, the impact of concurrent removal was minimal, likely due to the differences in surgical wounds locations and the distance between the two approaches. Conversely, this relationship was less clear for medial retropharyngeal and ilio‐sacral lymphadenectomies, as a high percentage of dogs experiencing postoperative complications also underwent mandibular (100%) or inguinal (74%) lymphadenectomy, respectively, compared to those not receiving concurrent superficial lymphadenectomy (0% and 38%). The authors suggest that additional surgical trauma from identifying and dissecting superficial LNs, along with longer surgical times, may have increased the risk of postoperative incisional complications, when using the same surgical access.^1^, ^33^ This hypothesis is reinforced by previous human metanalysis on pelvic lymphadenectomies.^9^, ^27^, ^29^ In humans, risk factors include the increased number of LNs excised, higher body mass index, and longer surgical times.^8^, ^27^, ^29^ Unfortunately, no risk factors associated with the development of postoperative complications were identified in this study. The only difference was found in follow‐up time, which was mildly but significantly longer in subjects that developed postoperative complications, both overall and in the axillary group, likely due to the increased need for long‐term patient monitoring by the clinician.

Mast cell tumors, carcinomas and melanomas were prevalent in our study population, likely due to their aggressive nature and higher metastatic potential. In these patients, regional or sentinel lymphadenectomy was often required as part of diagnostic and therapeutic protocols.^2^, ^19^, ^34^, ^35^ In cases such as soft tissue sarcomas and plasma cell tumors, lymphadenectomy is not routinely performed at our institution. However, it was selectively undertaken in specific cases where nodal metastatic involvement could have significantly impacted the patient's overall prognosis or when a definitive preoperative diagnosis was unavailable. In each of these instances, and following a general principle, the decision to perform lymphadenectomy was discussed during multidisciplinary rounds and with owners, weighing the pros and cons. The metastatic rates observed for various neoplasms aligned with findings from previous literature.^1^, ^2^, ^20^, ^34^, ^35^, ^36^

Many of the limits of this study stemmed from its retrospective design, particularly regarding the lack of standardized surgical plans and stratified postoperative medications and adjuvant treatments, as well as the involvement of different surgeons and the execution of additional procedures, which may have influenced the development of complications. Moreover, the concurrent and not standardized extirpation of superficial LNs may have impacted the assessment of complications. Additionally, our selection of medial retropharyngeal, axillary and ilio‐sacral LNs for analysis in the present research was arbitrary, based on institutional data and the frequency of removal in cases of skin or subcutaneous tumors. Other deep or intracavitary LNs could have been analyzed (i.e., sternal, parotid) but were excluded due to the low sample size. Therefore, further studies may help in definition of risk factor for intra‐ and postoperative complications after LN extirpation. Moreover, the absence of a power analysis may have partially influenced the ability to identify risk factors. Finally, the follow‐up period of at least 1 month might not have been sufficient to capture all long‐term complications.

In conclusion, lymphadenectomy of medial retropharyngeal, axillary, and ilio‐sacral LNs appears to be a generally safe procedure, with rare and mostly minor complications that are self‐limiting. However, ilio‐sacral lymphadenectomy via traditional caudal laparotomy may carry a higher risk of intraoperative complications, particularly in cases of enlarged nodes, as well as postoperative incisional complications, due to the intracavitary location of the LNs and to surgical approach used. The concurrent extirpation of superficial LNs may also affect the rate of postoperative incisional complications.

## AUTHOR CONTRIBUTIONS

Ciammaichella L, DVM: Cases management, manuscript preparation, data collection, data assessment, statistical analysis and editing. Campanerut J, DVM: Cases management, manuscript preparation, data collection and data assessment. Pisoni L, DVM, PhD: Cases management, data assessment and manuscript review. Cola V, DVM, PhD: Cases management, manuscript preparation and data assessment. Zanardi S, DVM: Cases management, data assessment and manuscript review. Foglia A, DVM, PhD: Cases management, data assessment and manuscript review. Ferrari C, DVM: Cases management, data assessment and manuscript review. Guerra D, DVM: Cases management, data assessment and manuscript review. Marconato L, DVM, DECVIM (Companion Animals): Cases management, data assessment and manuscript review. Del Magno S, DVM, PhD, DECVS: Study design, cases management, data assessment and manuscript review. All authors provided a critical review of the manuscript and endorsed the final version. All authors are aware of their respective contributions and have confidence in the integrity of all contributions.

## CONFLICT OF INTEREST STATEMENT

The authors declare no conflicts of interest related to this study. The authors have no financial disclosures to report.

## Data Availability

The data that support the findings of this study are available from the corresponding author upon reasonable request.
